# Reducing Protein Content with and Without Yeast Probiotic Actisaf Sc 47 Supplementation in the Diet of Dairy Cow: Effects on Nitrogen Use, Digestibility, and Rumen Microbial Protein

**DOI:** 10.3390/ani16081277

**Published:** 2026-04-21

**Authors:** Nizar Salah, Brigitte Gestes, Pauline Ly, Axel Blancou, Kheira Hadjeba, Julie Schulthess, Julie Duclos, Eric Pinloche

**Affiliations:** 1 Phileo by Lesaffre, 59520 Marquette-lez-Lille, France; b.gestes@phileo.lesaffre.com (B.G.); p.ly@phileo.lesaffre.com (P.L.); k.hadjeba@phileo.lesaffre.com (K.H.); j.schulthess@phileo.lesaffre.com (J.S.); j.duclos@phileo.lesaffre.com (J.D.); e.pinloche@phileo.lesaffre.com (E.P.); 2 Chambre d’Agriculture-Haute-Garonne, 31300 Toulouse, France; blancou.ax@gmail.com

**Keywords:** yeast probiotic, Actisaf Sc 47, dietary crude protein, nitrogen use, digestibility

## Abstract

This study aimed to evaluate the effects of lowering crude protein (CP) levels in the diet of dairy cows alone or in combination with a yeast probiotic on nitrogen utilization, digestion, and rumen microbial protein synthesis. In total, six cows were included in a 3 × 3 Latin square design. Each study period consisted of 23 days of diet adaptation and 5 days for sampling and data collection. Cows were randomly assigned to one of the three diets according to protein level and supplementation: control diet with 16.5 CP%DM (CTR), a diet with 14.5 CP%DM without Actisaf Sc 47 supplementation (LCP), and a diet with 14.5 CP%DM with Actisaf Sc 47 supplementation at 5 g/cow/day (LCPActisaf). Reducing protein content from 16.5 to 14.5 CP%DM alone or in combination with Actisaf Sc 47 significantly reduced nitrogen intake. Regarding fecal nitrogen (FN), no difference was observed between the CTR and LCP groups. However, FN was significantly lower in the LCPActisaf group compared with that of the CTR group. There was a tendency for lower FN in the LCPActisaf group compared with the LCP group. Compared with CTR, urinary nitrogen (UN) was not impacted by LCP but tended to decrease with LCPActisaf (*p* = 0.1). Compared with CTR and LCP, supplementation with Actisaf Sc 47 increased nitrogen use efficiency (NUE) (*p* < 0.05). A tendency for higher NUE was observed in the LCP group compared with the CTR group (*p* = 0.07). Crude protein digestibility was similar between the CTR and LCP groups and tended to increase with Actisaf Sc 47 supplementation. Compared with CTR, neutral detergent fiber (NDF) digestibility was not impacted by LCP but increased by LCPActisaf (*p* = 0.05); LCPActisaf did not impact NDF digestibility compared with LCP. Organic matter (OM) digestibility was similar among treatments. Although there was no difference between the three groups on rumen microbial protein synthesis (RMP), supplementation with Actisaf Sc 47 increased RMP. Reducing protein content in combination with Actisaf Sc 47 appears to be a good strategy, enabling both the environmental footprint linked to nitrogen losses to be reduced, and fiber digestibility and rumen microbial protein synthesis to increase.

## Simple Summary

Protein level in the diet of dairy cows is a key factor impacting production performance, efficiency, economic gain, and the environment. Lowering protein content is a strategy used to increase nitrogen use efficiency and decrease nitrogen losses; however, it can also impair milk production if not well controlled. Yeast probiotics could be used to mitigate the potential negative effects of decreased protein level on milk quantity and quality through the optimization of the rumen environment and fermentation. This study evaluated the effects of low-protein diets alone or in combination with yeast probiotic *Saccharomyces cerevisiae* (CNCM I-4407, 10^10^ CFU/g, Actisaf Sc 47; Phileo by Lesaffre, France) on nitrogen utilization (nitrogen intake, fecal nitrogen, urinary nitrogen, retained nitrogen, and nitrogen use efficiency), digestibility (crude protein, neutral detergent fiber, and organic matter), and rumen microbial protein synthesis trough allantoin, uric acid, and purine derivatives in dairy cows. Cows were fed a diet with crude protein levels of 16.5 CP%DM as the control (CTR), 14.5 CP%DM alone (LCP), or 14.5 CP%DM in combination with yeast probiotic Actisaf Sc 47 (LCPActisaf).

## 1. Introduction

Today, dairy farmers face multiple challenges, including improving economic efficiency and reducing their environmental footprint [1]. To address these, farmers must adopt integrated management strategies, among which nutrition, particularly protein nutrition, plays a pivotal role. Indeed, protein level directly influences animal performance, economic returns, and environmental outcomes. From an environmental perspective, the contribution of dietary protein to pollution is well documented, largely due to low nitrogen use efficiency (NUE) [2]. For instance, only 26 ± 38.2% of the nitrogen ingested by dairy cows is utilized for milk production; the remainder is primarily excreted in urine and feces [3]. A large-scale Canadian study involving 297,000 cows reported that 73.5% of ingested nitrogen is excreted, corresponding to approximately 12,000 tons of nitrogen per year [4]. Such low efficiency has significant environmental repercussions.

Nitrogen excreted in urine and feces contributes substantially to the following: the formation of nitrous oxide (N_2_O), a greenhouse gas with a global warming potential 298 times greater than CO_2_ and 10 times greater than methane [5,6]; the deterioration of air quality via ammonia (NH_3_) emissions; and the contamination of water resources through nitrate (NO_3_^−^) and nitrite (NO_2_^−^) leaching [7,8]. To mitigate these environmental impacts and develop more efficient, sustainable production systems, it is of paramount importance to improve NUE [9]. One promising strategy is to reduce the crude protein (CP) content of dairy cow rations. Several studies have demonstrated that lowering dietary CP significantly decreases urinary nitrogen (UN) excretion; for example, CP has been reduced from 18.4% to 15.1% [10], 20.1% to 16.2% [11], 16.7% to 14.8% [12], and 17.5% to 14.9% [13].

Although reducing CP content appears to be an interesting strategy, it must be implemented cautiously to maintain optimal balance among productivity, efficiency, and environmental stewardship because limited crude protein diets can impair rumen microbial growth and fermentation, potentially compromising both animal performance and environmental outcomes [14]. A potential strategy to mitigate risks associated with CP reduction involves enhancing rumen fermentation efficiency, specifically by reducing the proteolysis of dietary proteins and increasing the supply of rumen-undegraded protein (RUP) to the small intestine [15]. Yeast probiotics represent a feed additive capable of supporting these mechanisms, as evidenced by prior research [16,17]. To the best of our knowledge, no study has yet evaluated the combined effects of dietary CP reduction and yeast probiotic supplementation on dairy cow performance, with a specific focus on nitrogen utilization, nutrient digestibility, and rumen microbial protein synthesis, key factors that may influence milk production. Accordingly, this study was designed to address this knowledge gap.

## 2. Materials and Methods

### 2.1. Animals, Experimental Design, and Diets

An experiment was conducted at the Phileo by Lesaffre research station (The Farm, Chemin de Vallesvilles, Seysses, F-31600, France) between December 2024 and May 2025. All animal procedures strictly adhered to European Union guidelines on the protection of animals used for scientific purposes (Directive 2010/63/EU). The experimental protocol received approval from the French Ethical Committee for Animal Experimentation, Animal Sciences and Health N°115.

**Figure 1:**
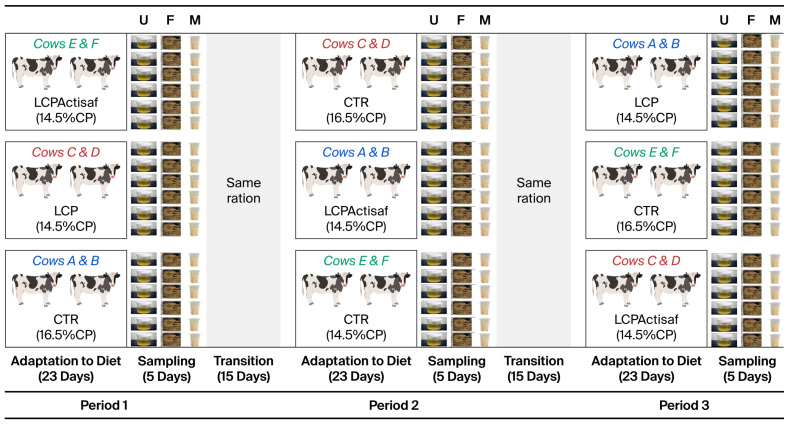
Experimental design and sampling. U = urine; F = feces; M = Milk; LCP: low crude protein without Actisaf (14.5% CP); CTR: control without Actisaf (16.5% CP); LCPActisaf: low crude protein with Actisaf (14.5% CP).

The trial was designed and reported in accordance with the ARRIVE guidelines, covering study design, sample size estimation, inclusion criteria, randomization, blinding, outcome measures, animal characteristics, statistical methods, and result presentation. Six healthy, multiparous Holstein dairy cows in their third lactation were selected for this study (mean ± SD: 92.5 ± 2.59 days in milk; 690.8 ± 79.1 kg body weight; body condition score: 2.7 ± 0.3; milk yield: 38.7 ± 5.9 kg/d). Inclusion was validated by a veterinarian and members of the institutional animal welfare committee. Cows were housed in a free-stall barn equipped with individual feed intake monitoring systems, except during milking and sample collection. The study followed a 3 × 3 Latin square design across three experimental periods during which each group of cows was successively fed the three types of treatments. Each period comprised 23 days of dietary adaptation followed by 5 days of measurement and sampling. A 15-day transition period separated consecutive experimental periods, during which all cows received a common basal ration (16.5% CP, 25.3% starch, 34.4% NDF on a DM basis) to minimize carryover effects (Figure 1).

Cows were allocated to three treatment groups based on dietary crude protein (CP) level and *Saccharomyces cerevisiae* CNCM I-4407 (Actisaf Sc 47) supplementation: negative control group (CTR): 16.5 CP%DM, no Actisaf Sc 47 supplementation; positive control group (LCP): 14.5 CP%DM, no Actisaf Sc 47 supplementation; experimental group (LCPActisaf): 14.5 CP%DM with Actisaf Sc 47 supplementation. Actisaf Sc 47 was administered at 5 g/cow/day via top-dressing. Cows were allocated to groups based on milk yield, DIM, and body weight. To ensure balance, cows were rotated through the treatments such that each cow received each diet once over the three periods (e.g., cows starting on CTR in Period 1 moved to LCPActisaf in Period 2 and LCP in Period 3). Health and behavior were monitored daily, including checks for mastitis, lameness, and reductions in dry matter intake (DMI) or milk yield. Inclusion criteria required cows to weigh between 600 and 850 kg, have a BCS between 2.5 and 3.0, and show no signs of health issues such as mastitis or lameness. Exclusion criteria included a prolonged decrease in DMI (≥15% for 3 consecutive days), contamination of fecal samples with urine or blood, or contamination of urine samples with feces or blood. Fecal and urine sampling was limited to a maximum of 10 min per cow; if sampling exceeded this duration, the sample was not collected to minimize stress.

At the start of the trial, cows in the CTR group were 93 ± 2.42 days in milk with 38.64 ± 4.99 kg of milk, cows in the LCP group were 91 ± 1.41 days in milk with 38.41 ± 0.31 kg of milk, and cows in the LCPActisaf group were 93 ± 4.24 days in milk with 39.25 ± 9.48 kg of milk. During the measurement periods, cows were housed in tie stalls and milked twice daily. The barn was equipped with mobile feed carts to enable individual feeding. Diets were offered as total mixed rations (TMRs) comprising corn silage, protein concentrate, energy concentrate, alfalfa hay, and a mineral–vitamin premix (ingredients and chemical composition are detailed in Table 1). All cows received a basal ration formulated at 14.5% CP (DM basis). For the CTR group, an additional quantity of protein-rich concentrate was provided during milking to achieve a final dietary CP level of 16.5% (DM basis). Cows were fed once daily ad libitum, with feed allowances adjusted to target ~5% orts (refusals). Feed offered and refused was weighed daily to determine individual dry matter intake. Fresh water was available ad libitum throughout the study period.

### 2.2. Measurements and Sampling

Diets were offered once daily at 09:00 h. For each cow, the quantities of feed offered and refused were weighed every morning prior to feeding to determine individual daily dry matter intake. During each 5-day measurement period, samples of the total mixed ration (TMR) offered and of individual orts (refusals) were collected daily. Samples were dried at 60 °C for 48 h in a forced-air oven to determine dry matter (DM) content, ground through a 1 mm screen using a hammer mill, and stored at room temperature until further chemical analysis. Cows were milked twice daily at 07:00 h and 17:00 h, and the total milk yield was automatically recorded at each milking.

Determining nitrogen balance and nitrogen use efficiency traditionally requires total urine collection using balance cages or catheters; however, these methods are laborious and may compromise animal welfare. Therefore, during the measurement period (days 22, 23, 24, and 25), we employed a spot urine sampling technique as described in [18]. This method involves collecting urine at specific time points over four consecutive days by stimulating the area below the vulva. Six samples were collected to represent a 24 h cycle at the following intervals after diet distribution: 4, 8, 12, 4, 8, and 24 h. Specifically, samples were taken at 13:00 h on day 22; 01:00 h and 17:00 h on day 23; 05:00 h and 21:00 h on day 24; and 09:00 h on day 25. For each cow at each sampling time, 100 mL of urine was collected. From this volume, two 15 mL aliquots were taken and acidified with 2 mL of 10% sulfuric acid (15 mL urine + 2 mL acid) to prevent ammonia formation and volatilization. Samples were frozen at −20 °C for further analysis; one aliquot was used for nitrogen analysis, and the other for creatinine, allantoin, and uric acid analysis. Urine output was estimated (Equation (1)) based on a creatinine coefficient of 29 mg/kg BW/day [19]. The total nitrogen excretion was calculated by multiplying the total urine volume by the corresponding nitrogen concentration.

(1) $$Urine\;output\;(\frac{kg}{day})=\frac{creatinine\;coefficient\times BW\;(kg)}{\left(creatinine\;concentration\;(\frac{mg}{dl})\times 10\right)}$$

The estimation of microbial protein synthesis in the rumen (RMP) was based on allantoin and uric acid, which allows the calculation of purine derivatives (PD) and then rumen microbial protein according to the following equations (Equations (2)–(5)). The total amount of purine absorbed was calculated using the equation of the International Atomic Energy Agency [20].

(2) $$PD\;(\frac{mmol}{day})=Allantoin\;(\frac{mmol}{day})+uric\;acid\;(\frac{mmol}{day})$$

(3) $$PD=0.85\times Absorption\;of\;PD+(0.385\times {BW}^{0.75})$$

(4) $$Absorption\;of\;PD=\frac{PD-\left(0.385\times {BW}^{0.75}\right)}{0.85}$$

(5) $$RMP\;(\frac{g}{d})=\frac{Absorption\;of\;PD\times 70\times 6.25}{\left(0.116\times 0.83\times 1000\right)}$$

Here, 0.83 is the average value for microbial nucleic acid digestibility; 70 mg N/mmol = purine N content; 0.116 = 11.6/100 = the purine N/total N ratio in mixed rumen microbes; and BW^0.75^ = the metabolic body weight of the cows.

Concurrent with urine sampling, fecal samples (500 g) were collected individually via direct rectal grab. Six samples were collected to represent a 24 h cycle at the following intervals after diet distribution: 4, 8, 12, 4, 8, and 24 h. Specifically, sampling occurred at 13:00 h on day 22; 01:00 h and 17:00 h on day 23; 05:00 h and 21:00 h on day 24; and 09:00 h on day 25. Each sample was oven-dried at 60 °C for 72 h in a forced-air oven to determine dry matter (DM) content, ground through a 1 mm screen using a hammer mill and stored in 150 g plastic containers until further analysis. To ensure consistent drying times and avoid bias, all samples were removed from the oven simultaneously. After drying, samples from each cow were pooled, hand-mixed, and a representative 100 g subsample per cow was sent to the laboratory for analysis. Acid-insoluble ash (AIA) was used as an internal marker to estimate apparent digestibility, as described in [21]. Rations and fecal samples were analyzed for identical constituents to estimate nutrient digestibility and total fecal output using the following equations:

(6) $$Nutrient\;digestibility\;(\%)=100\;(1-\frac{\%AIA\;diet}{\%AIA\;feces}\times \frac{\%\;nutrient\;feces}{\%\;nutrient\;diet})$$

(7) $$Fecal\;output\;(\frac{kg}{d})=\frac{AIA\;(\frac{g}{kg}DM)\times Dry\;matter\;intake\;(\frac{kg}{d})}{AIA\;in\;feces\;(\frac{g}{kg})}$$

### 2.3. Statistical Analysis

**Figure 2:**
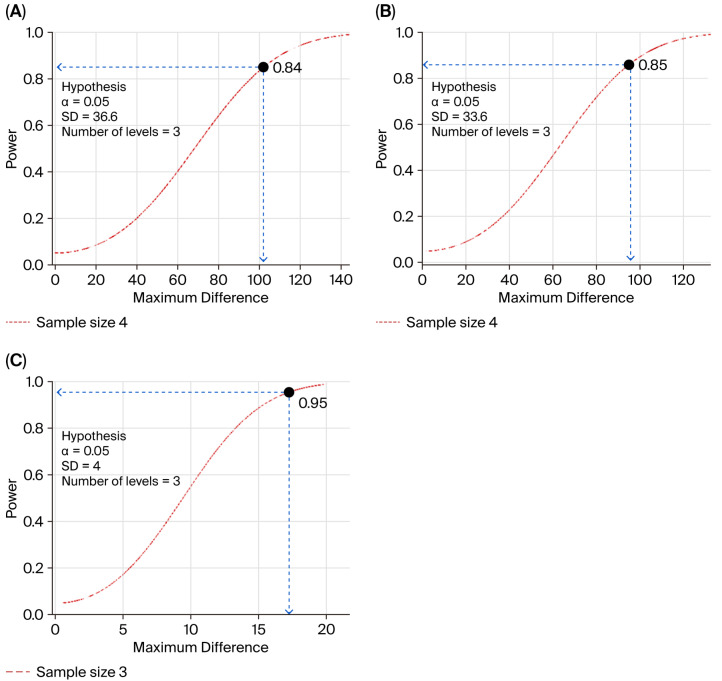
Power curve to estimate sample size according to UN (**A**), FN (**B**), and CP digestibility (**C**).

Power analysis was performed to determine sample size requirements for the primary response variables using previously published data [17,22]: fecal nitrogen (FN), urinary nitrogen (UN), crude protein (CP) digestibility, and rumen microbial protein synthesis [23,24]. At α = 0.05 and power = 0.80, the analysis indicated 4 cows were needed for UN and FN, and 3 cows for CP digestibility (Figure 2). The design was underpowered for rumen microbial protein synthesis. Due to facility limitations, 2 cows per treatment were used in a Latin square design.

The averaged data of the experiment were tested for normality using the Shapiro–Wilk test and then analyzed using a mixed-effects model in Minitab statistical software (version 19/ “C:\Program Files\Minitab\Minitab 19\”). The statistical model included treatment, period, and their interaction as fixed effects, and cow as a random effect. Data were analyzed using the PROC MIXED procedure as follows:

Y_ijk_ = µ + T_i_ + P_j_ + T_i_P_j_ + C_k_ + e_ijk_

where Y_ijk_ is the explained variable; µ, the general mean; T_i_ = fixed effect of treatment (CP level with and without Actisaf Sc 47 supplementation); P_j_ = fixed effect of the period (P1, P2 and P3); T_i_P_j_ = fixed effect of the interaction between diet treatment and period; C_k_ = the random effect of the cow; and e_ijk_ = the residual error.

An unstructured covariance matrix was selected based on the lowest Akaike Information Criterion (AIC) value. Outliers were identified using boxplots (values beyond ±1.5 × interquartile range) and the 95% confidence interval of normality probability plots. Statistical significance was set at *p* < 0.05, with tendencies noted at 0.05 ≤ *p* < 0.10. When treatment effects were significant or showed a tendency, means were compared using Tukey’s pairwise post hoc test. Each individual cow served as the experimental unit.

## 3. Results

### 3.1. Effect of Actisaf Sc 47 on Nitrogen Use

Nitrogen utilization data are presented in Table 2. Compared with the CTR group, nitrogen intake (NI) was reduced by both LCP and LCPActisaf (−12.95% and −17.44%, respectively; *p* = 0.003). Compared with the CTR group, FN was not impacted by LCP but was decreased by LCPActisaf (*p* = 0.04); LCPActisaf tended to decrease FN compared with LCP (*p* = 0.1). Compared with the CTR control, reducing protein alone did not impact urinary nitrogen, but reducing protein in combination with Actisaf Sc 47 tended to decrease UN (*p* = 0.1). No difference was observed between LCP and LCPActisaf. Retained nitrogen was not affected by treatment (*p* = 0.72). Compared with the CTR group, nitrogen use efficiency tended to increase with LCP and was significantly improved with LCPActisaf (*p* = 0.007).

### 3.2. Effect of Actisaf Sc 47 on Digestibility

Digestibility data are presented in Table 3. Crude protein (CP) digestibility did not differ between the CTR group and either of the LCP or LCPActisaf groups (64.3%, 63.5%, and 67.9% for CTR, LCP, and LCPActisaf, respectively); however, CP digestibility tended to be higher in LCPActisaf compared with LCP (*p* = 0.09). Relative to CTR and LCP, Actisaf Sc 47 supplementation increased CP digestibility by 5.6 and 6.9%, respectively. Neutral detergent fiber (NDF) digestibility was not affected by LCP compared with the CTR group, but was increased by LCPActisaf (*p* = 0.05). Reducing dietary CP without and with Actisaf Sc 47 supplementation increased NDF digestibility by 15% and 27%, respectively, compared with the CTR group. No significant difference was observed between LCP and LCPActisaf for NDF digestibility, although Actisaf Sc 47 numerically increased digestibility by 11%. Organic matter (OM) digestibility was not affected by treatment but was numerically higher in LCPActisaf and LCP compared with CTR (66.87%, 64.05%, and 61.51% for LCPActisaf, LCP, and CTR, respectively; *p* = 0.22).

### 3.3. Effect of Actisaf Sc 47 on Urinary Purine Derivates and Rumen Microbial Protein

Urinary purine derivative (PD) excretion and rumen microbial protein (RMP) synthesis data are presented in Table 4. Allantoin and uric acid excretion were unaffected by treatment (*p* = 0.80 and *p* = 0.70, respectively). Total urinary PD excretion did not differ among treatments (*p* = 0.96), and the estimated rumen microbial protein synthesis was similar between CTR and both LCP and LCPActisaf (*p* = 0.95).

## 4. Discussion

Increasing animal performance while reducing environmental impact represents one of the greatest challenges in dairy farming [25]. Nitrogen nutrition is a key factor that can be used to address this challenge, as it directly affects animal performance, economic returns, and environmental footprint [2]. Lowering dietary protein levels in dairy cows is a strategy employed to reduce nitrogen losses and increase nitrogen use efficiency. However, this approach must be used with caution, as excessive protein reduction can inhibit microbial growth and fermentation in the rumen, potentially compromising animal performance [14,15]. To achieve an optimal balance between performance and environmental sustainability while reducing dietary protein content, yeast probiotic supplementation appears to be a promising approach due to its beneficial effects on the rumen environment and fermentation. To the best of our knowledge, the effects of protein reduction in combination with the yeast probiotic Actisaf Sc 47 on nitrogen utilization and rumen microbial protein synthesis have not been previously investigated. Thus, our study aimed to investigate the effect of lowering protein level alone or in combination with a yeast probiotic on nitrogen use, digestibility, and rumen microbial protein.

Reducing crude protein (CP) from 16.5% to 14.5% of dry matter (DM), with and without Actisaf Sc 47 supplementation, decreased nitrogen intake (NI), and our results are in agreement with those of previous studies. For instance, the authors of [26] compared three protein levels and reported a positive correlation between dietary protein content and NI, noting that NI decreases with reduced nitrogen content in the ration. Similar findings were reported in [15], where lower NI was observed in cows fed 15.5% protein rations with and without essential oil supplementation compared to cows fed 16.5% protein rations. In this study, cows consumed similar quantities of feed across treatments; therefore, the decrease in NI can be attributed to the reduction in dietary CP content.

Dietary CP level is a predominant determinant of nitrogen excretion through urine and feces. Fecal nitrogen (FN) was not affected by lowering protein level alone but was reduced with Actisaf Sc 47 supplementation. The mechanisms by which Actisaf Sc 47 may reduce FN could be attributed to its positive effects on rumen fermentation and digestibility, as undigested nitrogen from endogenous or feed sources is excreted in feces [27].

The effect of reducing dietary crude protein (CP) on urinary nitrogen (UN) excretion is well documented, with numerous studies confirming a positive correlation between dietary CP content and UN excretion in dairy cows [28,29], sheep [30,31], and beef cattle [32,33]. Although this effect did not reach statistical significance in our study, lowering dietary CP from 16.5% to 14.5% DM without Actisaf Sc 47 supplementation reduced UN excretion by 21%. This finding aligns with [34], which reported a 21.6% reduction in UN when dietary CP was decreased from 16.2% to 14.4%. A similar trend was also observed in [15]. Actisaf Sc 47 supplementation tended to reduce UN excretion. Specifically, combining CP reduction with Actisaf Sc 47 supplementation decreased UN by 25%. This reduction may be attributed to lower ruminal NH_3_-N concentrations resulting from the combined effect of dietary CP restriction and yeast supplementation, as previously reported [35]. The observed decrease in urinary nitrogen losses can be explained by Actisaf’s ability to stimulate the growth and metabolic activity of fibrolytic bacteria in the rumen [17,36]. These microorganisms utilize ammonia as a nitrogen source, thereby enhancing microbial protein synthesis and overall ruminal nitrogen efficiency, which subsequently reduces UN excretion [37,38]. By lowering UN excretion, a major contributor to ammonia and nitrous oxide emissions, Actisaf Sc 47 supplementation may help mitigate environmental pollution [39]. Recent life cycle assessment studies [40,41] have demonstrated that Actisaf Sc 47 reduces the carbon footprint and associated environmental impacts in dairy and beef production systems. Given the well-documented adverse effects of ammonia on animal health, reproduction, and welfare, as well as on human health [42], we hypothesize that Actisaf Sc 47 supplementation may indirectly mitigate these issues by improving nitrogen utilization and reducing nitrogenous emissions.

Several studies have reported low nitrogen use efficiency (NUE) in dairy cows [43]. For instance, the authors of [3] documented mean NUE values of 25% in North America and 28% in Northern Europe. Various strategies have been explored to improve NUE, including reducing dietary crude protein (CP) concentration. In our study, we observed that reducing dietary CP by 2% improved NUE, and this improvement was significantly more pronounced when cows were supplemented with Actisaf Sc 47. The 2% CP reduction increased NUE by 10%, which aligns with the findings of [26]: a 9% increase in NUE following dietary CP reductions of 1% and 2%. Similarly, other studies have demonstrated that reducing CP by 1% and 2.9% improves NUE by 11% and 16%, respectively [15,29]. The improvement in NUE was more pronounced with Actisaf Sc 47 supplementation. Specifically, reducing dietary crude protein in combination with Actisaf Sc 47 increased NUE by 24%. The biological mechanisms underlying this effect remain unknown and have not been previously described. We hypothesize that Actisaf Sc 47 enhances NUE through multiple pathways. First, it may decrease the proliferation of proteolytic bacteria, such as *Prevotella ruminicola* [44]. Second, Actisaf Sc 47 may increase the abundance and activity of cellulolytic bacteria, which primarily utilize NH_3_-N as their nitrogen source [45]. Third, Actisaf Sc 47 may reduce blood urea nitrogen (BUN) concentrations, which are positively correlated with ruminal NH_3_-N levels. The reduction in blood urea nitrogen concentration implicates a possible mechanism of higher nitrogen capture ability and its conversion to microbial protein and, consequently, better NUE [46]. The relatively large standard error of the mean (SEM) observed in this study may be attributed to the limited number of animals and considerable inter-animal variability, consistent with our power analysis findings. Nevertheless, these results suggest that supplementing Actisaf Sc 47 in a low-protein diet represents a promising nutritional strategy to reduce nitrogen losses and enhance nitrogen use efficiency.

In ruminants, 50–80% of the protein absorbed in the small intestine originates from rumen microbial protein synthesized within the rumen [47]. Estimating microbial protein is crucial for diet formulation and NUE and nitrogen losses to the environment [48]. Microbial protein synthesis is influenced by multiple factors, including physical, chemical, biological, endogenous, and dietary variables, with dietary factors exerting the predominant influence. Key dietary factors include feed type and inclusion level, forage quality, and supplementation with feed additives such as yeast-based probiotics [49,50]. For a long time, the reference method for quantifying rumen microbial protein synthesis relied on animals fitted with ruminal and post-ruminal cannulas—a costly, invasive, and impractical technique under farm conditions [51]. Increasing emphasis on animal welfare has spurred the development of non-invasive, field-applicable alternatives, such as measuring the urinary excretion of purine derivatives (e.g., allantoin and uric acid). In this study, we employed urinary purine derivative excretion to estimate rumen microbial protein synthesis. Reducing dietary crude protein from 16.5% to 14.5% without Actisaf Sc 47 supplementation had no significant effect on urinary allantoin, uric acid, or total purine derivative concentrations. These findings align with previous reports on dairy cows [15] and Murrah buffaloes [52]. To the best of our knowledge, this is the first study to investigate the combined effects of dietary protein reduction and yeast probiotic supplementation on the urinary excretion of purine derivatives (allantoin and uric acid) and estimated rumen microbial protein synthesis. Although no statistically significant differences were observed among the three treatment groups, cows fed the 14.5% CP diet (DM basis) supplemented with Actisaf Sc 47 exhibited numerically higher microbial protein synthesis. This effect may be attributed to the beneficial effects of Actisaf Sc 47 on the ruminal environment and microbial metabolism. By stabilizing ruminal pH and scavenging oxygen, the probiotic likely enhanced fiber degradation and substrate availability, thereby optimizing microbial activity and promoting microbial protein synthesis [53,54]. A limitation of this study is the small sample size (*n* = 6), which may have reduced the statistical power required to detect significant differences in these estimated parameters. Nevertheless, the present methodology and findings provide a solid foundation for future large-scale trials with increased statistical power. During our study, we used the spot urine sampling technique as described by [18], which potentially limits the accuracy and the precision estimation of our results compared to the total collection of urine. The high variation explained by the standard deviation of the mean could be explained first by the low number of animals and the indirect sampling method.

Reducing dietary crude protein without Actisaf Sc 47 supplementation did not affect crude protein digestibility in this study. This finding contrasts with those of [31], which reported a decline in CP digestibility when dietary protein content was reduced from 16.2% to 14.4%. The absence of a significant response in CP digestibility between the 16.5% and 14.5% CP diets (DM basis) without Actisaf Sc 47 supplementation might suggest that rumen-degradable protein supply at the 14.5% CP level was sufficient to maintain rumen microbial activity [29]. However, supplementation with Actisaf Sc 47 at the 14.5% CP level tended to increase CP digestibility, which aligns with previous findings in dairy cows [55,56] and beef cattle [57]. The potential mechanism by which Actisaf Sc 47 enhances crude protein (CP) digestibility can be attributed to improved ammonia uptake to support microbial metabolic activity, thereby increasing microbial efficiency, as suggested in [58]. Regarding neutral detergent fiber (NDF) digestibility, reducing dietary protein content without Actisaf Sc 47 supplementation had no significant effect. This finding aligns with [34], who reported marginally higher NDF digestibility with a 16.2% CP diet compared to diets containing 14.2% or 13.4% CP. Supplementation with Actisaf Sc 47 tended to increase NDF digestibility. This positive effect may be attributed to the probiotic’s ability to stimulate the growth and enzymatic activity of fibrolytic and cellulolytic bacteria [36,58], thereby enhancing fiber degradation. The variability in responses to crude protein (CP) reduction across studies may be attributed to methodological differences in digestibility assessment, including total fecal collection versus spot sampling, the use of direct measurement techniques versus indigestible markers, stage of lactation, and ration composition. A limitation of the present study is the small sample size (*n* = 6), which may have reduced statistical power to detect significant differences in these estimated parameters. Nevertheless, the methodology and findings presented here provide a foundation for future large-scale trials with increased animal numbers and enhanced statistical robustness.

## 5. Conclusions

Reducing dietary crude protein (CP) from 16.5% to 14.5% (DM basis) decreased nitrogen intake without affecting fecal, urinary, or retained nitrogen excretion and tended to improve nitrogen use efficiency (NUE). Compared with the control diet (16.5% CP), supplementation with Actisaf Sc 47 at the 14.5% CP level reduced fecal nitrogen, tended to reduce urinary nitrogen, and significantly increased NUE. Relative to the non-supplemented 14.5% CP diet, Actisaf Sc 47 supplementation did not alter nitrogen intake or urinary nitrogen, tended to decrease fecal nitrogen, and significantly enhanced NUE. The 2-percentage-unit reduction in CP alone had no effect on the digestibility of CP, organic matter (OM), or neutral detergent fiber (NDF). However, Actisaf Sc 47 supplementation at 14.5% CP increased NDF digestibility compared with the control and tended to improve CP digestibility relative to the non-supplemented 14.5% CP diet, without affecting OM or NDF digestibility. Neither CP reduction nor Actisaf Sc 47 supplementation significantly altered estimated rumen microbial protein synthesis, although numerically higher values were observed in the LCPActisaf group. In conclusion, combining dietary CP reduction with Actisaf Sc 47 supplementation represents a promising strategy for optimizing nitrogen utilization, minimizing nitrogen excretion, and supporting more sustainable dairy production systems. Future large-scale studies with increased sample sizes are warranted to validate these findings and refine dietary formulations for practical application.

## Figures and Tables

**Table 1:** Composition and nutritional values of diets.

Groups	CTR (16.5% CP)	LCP (14.5% CP)	LCPActisaf (14.5% CP)
Composition of the Diet (%DM)			
Corn silage	64.6	68.1	68.1
Alfalfa hay	9.5	10.0	10.0
Energy concentrate	8.0	8.5	8.5
Protein concentrate	12.5	13.2	13.2
Protein concentrate ^1^	5.2		
Mineral	0.2	0.2	0.2
Actisaf Sc 47 ^2^ (g/d)			5
Nutritional Values			
DM (%)	48.10	46.80	46.80
CP (%DM)	16.50	14.50	14.50
NDF (%DM)	37.40	38.20	38.20
ADF (%DM)	19.10	19.40	19.40
Crude fiber (%DM)	18.40	18.90	18.90
UFL (/kgDM)	0.875	0.874	0.874
PDIE (g)	103	95.70	95.70
PDIN (g)	108	94.50	94.50
Starch (%DM)	25.3	26.70	26.70
Digestible lysine (%PDIE)	7.01	6.55	6.55
Digestible methionine (%PDIE)	1.97	1.85	1.85

^1^ Protein concentrate added during milking; ^2^ Actisaf Sc 47 mixed with 50 g of concentrate. CTR: control group (16.5% CP without Actisaf); LCP: 14.5% CP without Actisaf; LCPActisaf: 14.5% CP with Actisaf Sc 47); UFL: forage unit for lactation; PDIE: true protein absorbable in the small intestine when rumen fermentable energy is limiting microbial protein synthesis in the rumen; PDIN: true protein absorbable in the small intestine when degradable nitrogen is limiting microbial protein. synthesis in the rumen.

**Table 2:** Effect of reducing CP with and without Actisaf Sc 47 supplementation on nitrogen use.

	CTR	LCP	LCPActisaf	SEM	*p*-Value		
					T	P	T × P
NI (g/d)	652.0 ^a^	577.2 ^b^	551.3 ^b^	16.4	0.03	0.35	0.81
FN (g/d)	233.0 ^a^	211.2 ^a,b,^*	182.98 ^b,^*	13.8	0.04	0.19	0.53
UN (g/d)	264.4 ^a,^*	218.3 ^a,b^	211.0 ^b,^*	17.3	0.10	0.54	0.20
RN (g/d)	153.9 ^a^	147.6 ^a^	157.3 ^a^	19.06	0.71	0.34	0.20
NUE (%)	28.3 ^a,^*	31.3 ^a,^*	35.10 ^b^	2.45	0.007	0.07	0.47

^a,b^ Mean values in the same column with different superscripts differ significantly (*p* < 0.05). * Mean values in the same column with a significant difference tendency (*p* < 0.1). CTR: control without Actisaf (16.5% CP); LCP: low crude protein without Actisaf (14.5% CP); LCPActisaf: low crude protein with Actisaf (14.5% CP); SEM: standard error of the mean; T: treatment effect; P: period effect; T × P: treatment and period interaction effect; NI: Nitrogen intake, FN: fecal nitrogen; UN: urinary nitrogen; NUE: Nitrogen use efficiency (N in milk/N intake) × 100; RN: retained nitrogen = NI − (UN + FN).

**Table 3:** Effect of reducing CP with and without Actisaf Sc 47 supplementation on nutrient digestibility.

	CTR	LCP	LCPActisaf	SEM	*p*-Value		
					T	P	T × P
Dig CP (%)	64.3 ^a^	63.5 ^a,^*	67.9 ^a,^*	2.29	0.09	0.12	0.59
Dig NDF (%)	31.7 ^a^	36.5 ^a,b^	40.6 ^b^	3.22	0.05	0.17	0.68
Dig OM (%)	61.7	64.0	66.9	1.70	0.22	0.31	0.85

^a,b^ Mean values in the same column with different superscripts differ significantly (*p* < 0.05). * Mean values in the same column with a significant difference tendency (*p* < 0.1). CTR: control without Actisaf (16.5% CP); LCP: low crude protein without Actisaf (14.5% CP); LCPActisaf: low crude protein with Actisaf (14.5% CP); SEM: standard error of the mean; T: treatment effect; P: period effect; T × P: treatment and period interaction effect.

**Table 4:** Effect of reducing CP with and without Actisaf Sc 47 supplementation on purine derivates excreation and rumen microbial synthesis (RMP).

	CTR	LCP	LCPActisaf	SEM	*p*-Value		
					T	P	T × P
Allantoin (mmol/d)	356.9	369.5	388.7	42.4	0.9	0.28	0.86
Uric acid (mmol/d)	22.02	32.88	29.11	8.19	0.7	0.15	0.13
Purine derivates (mmol/d)	378.9	402.4	417.8	46.9	0.96	0.25	0.84
RMP (g/d)	1748	1874	1957	234	0.95	0.25	0.85

SEM: standard error of the mean; T: treatment effect; P: period effect; T × P: treatment and period interaction effect; RMP: rumen microbial protein; CTR: control without Actisaf (16.5% CP); LCP: low crude protein without Actisaf (14.5% CP); LCPActisaf: low crude protein with Actisaf (14.5% CP).

## Data Availability

The data presented in this study are available within the manuscript.
